# The effect of a mindfulness intervention (MI) on sleep disturbance (SD) among nurses

**DOI:** 10.1038/s41598-024-55748-5

**Published:** 2024-03-01

**Authors:** Audai A. Hayajneh, Malak O. Al-Younis, Mohammad Rababa

**Affiliations:** Adult Health-Nursing Department, Faculty of Nursing, Jordan University of Science and Technology, P.O. Box: 3030, Irbid, 22110 Jordan

**Keywords:** Mindfulness intervention, Sleep disturbance, Nurses, Health policy, Quality of life

## Abstract

Sleep disturbance (SD) makes it difficult for nurses in intensive care units (ICUs) to perform activities that require focused and continual concentration, which raises the risk of medical errors, health issues, loss of sleep, and patient care mistakes. The mindfulness intervention (MI) was created to give participants the capacity to approach their own emotions with non-judgmental awareness and to become more conscious of their thoughts and feelings, and it reduced psychological symptoms. This study examined the effect of MI on SD among nurses. A randomized control trail (RCT) was conducted and recruited 100 nurses from intensive care and medical-surgical units from three hospitals located at the northern and middle regions of Jordan. Bivariate analysis including independent T-test and multiple linear regressions were used to study the differences between the interventional group (MI) and the comparison group (watching mindfulness videos) in terms of the impact on the SD. Nurses reported significant and high levels of SD. MI significantly reduced the level of SD and improved sleep quality among nurses. MI should be integrated into nursing competences to combat the negative impacts of poor sleep quality on nurses and organizational-sensitive outcomes.

## Introduction

Sleep disturbance (SD) is defined as a condition in which a person cannot get adequate sleep and receives less sleep than is essential for feeling refreshed^1^. The amount of sleep required to feel rested and perform properly varies by person and with age, but it is necessary to get adequate sleep each night because sleep provides our bodies a chance to be restored. Therefore, decreasing sleep might be harmful to health^2^.

SD among critical care nurses is not well acknowledged and is usually clumped under a wider umbrella of burnout and stress rather than a separate phenomenon of its own^3^. In the United States, the Critical Care Societies Collaborative^4^ acknowledged that nurses and other critical care healthcare professionals experience SD due to a number of factors including useless coping mechanisms, work-life imbalance, younger age, an insufficient support system, a lack of social assistance, a heavy workload, a lack of control over the type and quality of working conditions, insufficient benefits, and a lack of teamwork^4^.

According to Johnson and colleagues^1^, around half (56%) of nurses who worked in night shift reported being sleep unlike nurses who worked another shift. The primary causes of SD were decreased opportunities for sleep. Patients were exposed to a decreased care quality during night shifts ^1^. SD makes it challenging for nurses to complete tasks that require intense and continuous focus, thus leading to a decreased care quality ^5^. Furthermore, SD can lead to health issues for nurses such as a higher risk of obesity, diabetes mellites (DM), gastrointestinal problems, and heart diseases ^5^.

Nurses in intensive care units experience urgent and traumatic challenges as well as life-threatening situations; their patients require high levels of attention ^1^. Thus, SD needs to be reduced or properly prevented because it can decrease the quality of care ^1^. Moreover, SD causes impaired cognitive performance among nurses, which in turn leads to a low level of alertness ^6^ among nurses. The impact of SD is more pronounced among nurses working the night shift because they experience lower sleep quality and shorter sleep duration than those working the day shift ^7^.

Nurses can suffer from SD that results from life-threatening situations and traumatic events. Here, a mindfulness intervention (MI) can improve care quality among nurses in Jordanian hospitals, and thus developing an MI program for nurses might help to combat SD while collaborating with patients who suffer from traumatic events and critical conditions. Thus, this study examined the differences in SD among nurses assigned to an interventional group (MI) or a comparison group (watching mindfulness videos). This study also aims to identify the factors associated with SD.

## Methods

### Design

A randomized control trial (RCT) design was used in this study. A convenience sample of 100 participants was assigned equally and randomly to two groups: the interventional group (MI) and the comparison group (watching mindfulness videos).

### Setting and sampling

The participants were recruited from acute and critical care units at three hospitals located at the northern and middle regions of Jordan. The sample size was calculated using G power calculation. The results suggested a sample size of 50 participants for each group (the interventional group and the comparison group); the total sample size was thus 100 participants. The G power calculations used an effect size yield for generalized linear mixed models and the difference between two groups; these were 0.15 with a power of 0.8, which is suitable to measure the effect of each group. The inclusion criteria were: Jordanian nurses who: (1) at least hold a bachelor’s degree and (2) provide direct care to patients. The exclusion criteria were floating nurses who work in different departments and nurses who previously attended mindfulness workshops.

### Measures

Data about age, gender, level of education, marital status, spouse education, income, working status (variable or fixed shift), type of shift (A, B, C or day, night), experience (year), hours worked daily, hours worked weekly, and department (medical, surgical, intensive and critical, and emergency departments) were gathered from the nurses.

The Mindful Attention Awareness Scale (MAAS) has exhibited discriminant, convergent, known-groups, and criterion validity. The Cronbach’s alpha values ranged from 0.80 to 0.90, thus indicating acceptable levels of reliability^8^. The MAAS is a 15-item scale that was developed to assess one of the core components of mindfulness: a receptive state of mind, in which attention is directed by a sensitive awareness of what is occurring in the moment and simply observing what is occurring^8^. The 15-item trait version of the MAAS has a single factor structure and has items with scores ranging from 1 (almost usually), 2 (extremely frequently), 3, 4, and 5 (very occasionally) to 6 (nearly never). A higher mean score on the scale corresponds to higher levels of dispositional mindfulness^8^.

The Pittsburgh sleep quality index (PSQI) is a questionnaire that can evaluate sleep quality over one month. The first four questions are about bedtime, number of minutes required to fall asleep, time of waking, and hours of sleep. The next 10 questions ask about how often participants had trouble sleeping due to multiple causes (e.g. cough, bad dream, cold/hot, need to use the bathroom) and the use of sleep medications. These were answered over a four-point scale (never, less than once weekly, once or twice a week, or three or more times a week) ^9^. Other questions are rated from very good, fairly good, fairly bad, and toward very bad. The instrument also asks if the participant has had trouble maintaining enthusiasm for completing tasks. The response options were no problem, only very slight problem, somewhat of a problem, and very big problem. It has 19 items creating seven components, and each component ranged from 0 = no difficulty to 3 = severe difficulty. The total score is then summed and ranged from 0 to 21. A high total score indicates worse sleep quality^9^. Cronbach’s alpha indicates good internal reliability (0.7) and showed construct validity^10^ with a confirmatory factor analysis illustrating construct validity^11^.

### Data collection

The worked received the institutional review board (IRB) approval from Jordan University of Science and Technology (Ref. #20,230,141), approving this experiment, including any relevant details. All methods were carried out in accordance with relevant guidelines and regulations. The participants completed written consent form. Informed consent was obtained from all subjects and/or their legal guardian(s). Prior to the study, the investigator met with the manager of the intensive care unit and the medical-surgical units in each hospital that was chosen to coordinate the workflow of the study procedure, to discuss the time/date of the session, to choose the location for a session, and to collect data based on the schedule of the nurses participating in the study. After discussing the inclusion and exclusion criteria, the researcher obtained a list of nurses who would participate.

The researcher first gave a brief explanation of the study to the nurses before having them sign a consent form. During this time, the researcher remained in the same location to answer any questions. After giving all 100 nurses a consent form, the researchers divided the nurses randomly into two groups (the intervention group and comparison group). The intervention group received four sessions of mindfulness practices per month with one session per week. The protocol of this frequency for the mindfulness-based interventions was performed according to several studies that used approximately and similarly the same duration of frequency of MI sessions and then measured the effectiveness of MI on their main variables after four weeks^12–14^. Participants demonstrated significant improvements in the outcomes of their studies after using mindfulness-based interventions. The nurses who participated had a busy schedule of working bedside hours, and they attended MI sessions at a convenient time. This—along with alongside administrative barriers—might have led to the use of four-week duration MI sessions. This study used the MAAS questionnaire every week for one month due to the weekly change in self-reported mindfulness to measure improvements among the participants as suggested in a recent study^15^.

The MI sessions were conducted by an expert certified in psychotherapy, educational counseling, and MI. This expert and authors met the participants face-to-face in a hallway inside the hospital for one hour per session. The comparison group simply watched MI videos. The two groups completed the questionnaire before the 1st session and after the 2nd, 3rd, and 4th sessions. The researchers stayed with the participants for 20 min to clarify any misunderstanding of the questions.

### MI in both groups (Intervention Vs. Comparison)

MI was conducted in the intervention group over four weeks (Table 1). Participants in the comparison group were assigned to watch four different videos about mindfulness with one video per session (Table 2).

**Table 1:** The schedule of mindfulness intervention program for the interventional group.

Week#	Activity	Duration
1	Introduction about mindfulness and meditation Definition, how it works, how does the mind work with it, what is the effect on body and mind, benefits, Questions, and Answers 15 min for meditation exercise	1 h
2	Teaching how to do Breathing and Body scan Mindfulness meditation Teaching the 5 min mindful breathing technique Meditation exercise for 15 min	1 h
3	Teaching some mindfulness practices in daily life, and how much it is important to give ourselves time to relax and eliminate negative energy Meditation exercise for 15 min include breathing, body scan	1 h
4	Full session of Mindfulness meditation including dealing with thoughts and feeling An extended guided meditation that includes intention, breathing, relaxation, centering, observing thoughts and feeling in body parts, repeating words closing the session, sharing experience, question, and answers	1 h

**Table 2:** The schedule of videos for the comparison group.

Week#	Activity	Duration
1	Meditation of stress (guided meditation for stress release in a short version where participant will have a short body relaxation)	30 min
2	Meditation of stress (guided meditation for stress release in a short version where participant will have a short body relaxation then followed by concentration and releasing accumulated pressure during the day)	30 min
3	Meditation for deep sleep (This meditation will be used before sleeping as it is a golden hour the one before sleeping to help the participant to let go any thoughts from the day and to go into a deep sleep to reach the benefit of a good sleep for a fresh morning the next day)	30 min
4	Meditation for Loving Kindness (This meditation will help participant to be more compassionate with themselves and others as if practiced on daily basis they will notice the difference after that)	30 min

### Statistical analysis

The mean and standard deviation were measured for continuous variables and medians; IQR was used for continuous variables with statistical skewness. The frequency and percentage of categorically variables were measured. Chi-squared was used to see how much of a difference there was between different categorical variables. However, the LR (likelihood ratio)-corrected Chi-square was used if there were any statistical assumptions that were violated for Chi-square.

The generalized linear mixed model (GLMs) was used for repeated measurement data. Here, data about the PSQI represents sleep quality over the last month measured at baseline (pre-test) and at the end of the month (post-test) after completed four MI sessions (one per week). Regression analysis compared the mindfulness awareness score of the professional quality-of-life (PROQOL) questionnaire to each of the nurses in the intervention groups along with the interaction between intervention and time, nurses’ sociodemographic characteristics, and related working and professional factors. The association between predictor variables and the analyzed outcome was expressed as a multivariate adjusted beta (β) coefficient with a 95% confidence interval. SPSS statistical computing program (IBM, Version 27)^16^ was used for all statistical analyses with an alpha significance level of 0.05.

### Ethical considerations

IRB approval was obtained from Jordan University of Science and Technology (Ref. #20,230,141), approving this experiment, including any relevant details. All methods were carried out in accordance with relevant guidelines and regulations. Informed consent was obtained from all subjects and/or their legal guardian(s). The consent forms were signed by participants who are eligible and agreed to participate in the study. The participants had the right to withdraw from the study at any time without any consequences. Participants’ information was de-identified and not shared with anyone without authorization. Each had a certain number and deals with this number during data collection.

## Results

This study evaluated 100 nurses. Most nurses were female (69%), and around two-thirds (92%) had a bachelor's degree in nursing; 71% of the nurses were aged between 25 and 30 years, and the remaining were between 31 and 40 years old. A significant proportion (59%) of the nurses were never married. The study also looked at the educational attainment of the nurses with 92% having bachelor’s degrees and 8% having master's and doctoral degrees in nursing (Table 3).

**Table 3:** Descriptive analysis of the nurses sociodemographic characteristics and working and professional conditions (N = 100).

		Frequency (%)	Mean (SD)	Median (IQR)
Age (years)			29.76 (3.83)	
Age group	25–30 years	71 (71%)		
	31–40 years	29 (29%)		
Gender	Male	31 (31%)		
	Female	69 (69%)		
Marital state	Never married	59 (59%)		
	Ever married	41 (41%)		
Educational Level	Bachelor’s degree	92 (92%)		
	Master’s and doctoral degrees	8 (8%)		
Spouse educational level	Unmarried or have partner with High school or less education	70 (70%)		
	Bachelors	30 (30%)		
Households monthly Income (JOD)			486 (62.57)	
Sufficiency of HH income	Insufficient & indebt	25 (25%)		
	Insufficient	45 (45%)		
	Sufficient	30 (30%)		
Family size (members)				5 (2)
House size (number of rooms)				4 (1)
Worked shifts type	Fixed shifts	9 (9%)		
	Variable floating shifts	91 (91%)		
Shifts worked	ABC	83 (83%)		
	DAY-NIGHT	10 (10%)		
	Night shifts	7 (7%)		
Number of hours worked per shift	8-Hour shifts	83 (83%)		
	12-Hour shift	17 (17%)		
Total Number of hours worked per week	36 Hours	15 (15%)		
	40 Hours	83 (83%)		
	48 Hours	2 (2%)		
Clinical experience years			5.71 (3.26)	
Working department	Critical care units	60 (60%)		
	General hospital wards	40 (40%)		

%: Percent; SD: standard definition; JOD: Jordanian dinars; IQR: interquartile range; ABC: A shift, B shift, C shift.

**Figure 1:**
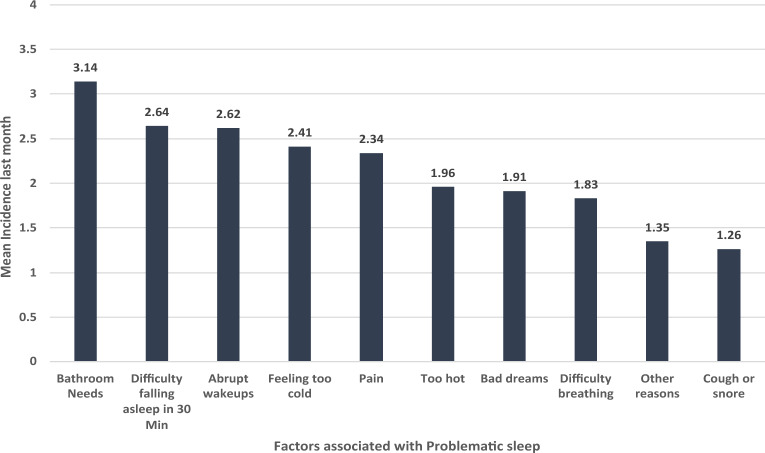
The Nurses baseline time mean perceived incidence of sleep offending factors last month.

The nurses were asked to rate the frequency of having sleep disturbance due to various reasons using a Likert scale graded from 1 (never in the last month) to 4 (three to four times in the last month). The findings revealed that the top sleep offender for nurses in the last month was the need to wake up for bathroom use (mean score = 3.14/4). The second-most common sleep-delaying factor reported by nurses was difficulty falling asleep within 30 min. Other factors included abrupt wakeups during sleep at night or early morning, feeling too cold, and experiencing pain that troubled their sleep. The least common issues were difficulty breathing while asleep, having bad dreams, feeling too hot while asleep, coughing, and snoring. See Fig. 1.

The nurses were also prompted to specify how often they had trouble staying awake while driving, eating meals, or engaging in social activities. The findings showed that 38% of the nurses did not experience these difficulties. However, 46% of them had trouble staying awake during activities less than once per week, and 16% of them had this problem once or twice per week in the past month. The nurses were also asked to rate their sleep quality over the past month: 28% of the nurses reported very good sleep, while 35% reported fairly good sleep quality. On the other hand, 12% reported fairly bad sleep quality, and 16% experienced very bad sleep quality.

### Mindfulness of both groups (Baseline Vs. The end)

**Figure 2:**
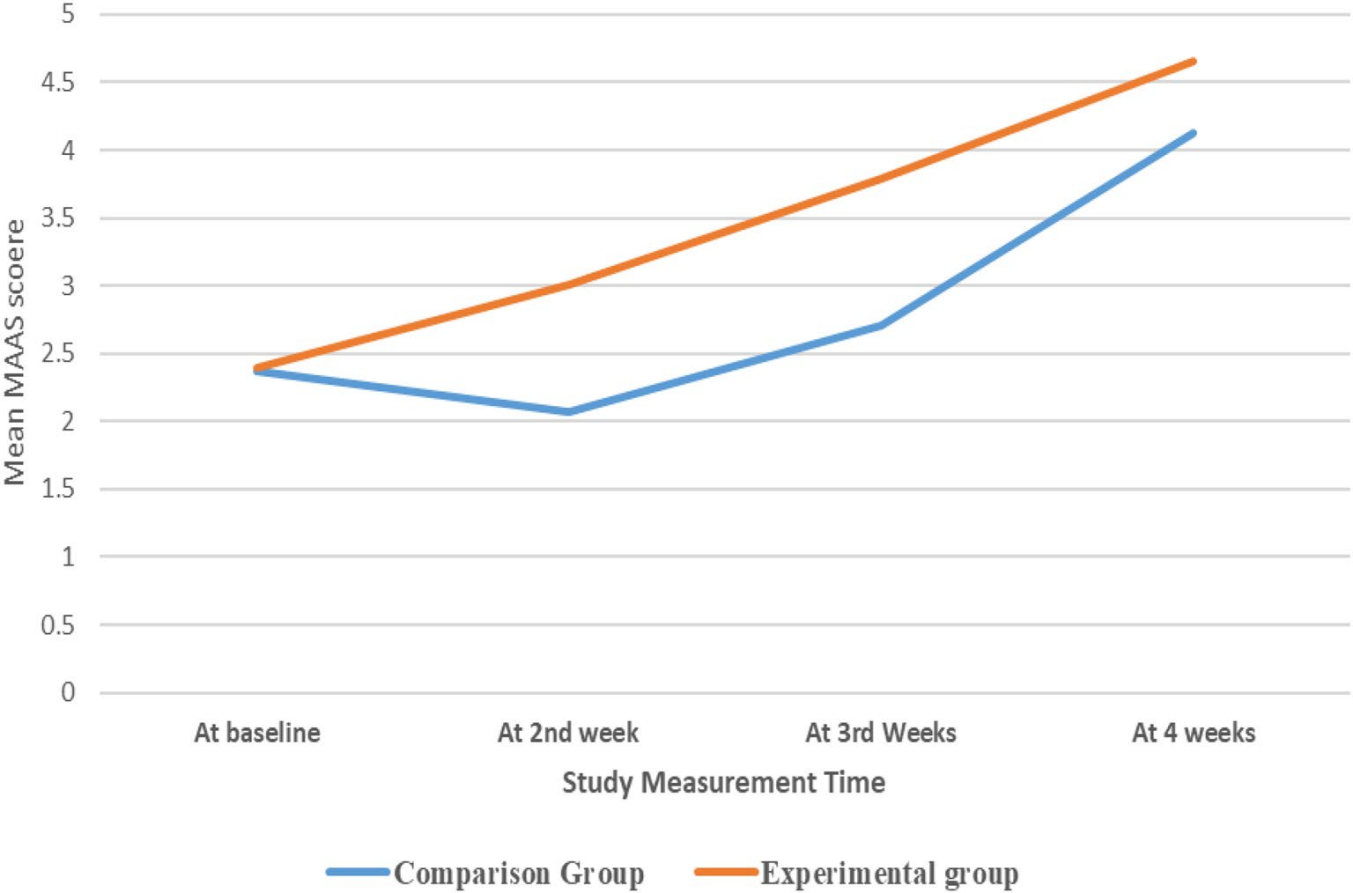
The nurses mean perceived Mindfulness Awareness across time for both study groups.

The analysis showed that, at baseline, the two groups (comparison vs. intervention) did not significantly differ in terms of their mean MAAS scores (*p*-value = 0.70) as shown in the Fig. 2. However, the analysis model revealed that all nurses, regardless of the groups they belonged to, had significantly higher MAAS scores at the fourth week compared to their baseline (beta coefficient = 1.138, *p*-value < 0.001). The nurses' mean perceived MAAS score at the second week was also significantly higher than at baseline in general (beta coefficient = 0.213, *p*-value = 0.003). However, the overall mean MAAS score at the first week and baseline did not differ significantly (*p*-value = 0.082).

The analysis model showed that the interaction effect of time * MI was statistically significant. The nurses who received mindfulness therapy recorded significantly higher mean MAAS scores at the fourth week relative to the comparison group (beta coefficient = 0.343, *p*-value = 0.006). Similarly, the intervention group had significantly higher mean MAAS scores at the third week compared to the comparison group of nurses (beta coefficient = 0.738, *p*-value < 0.001). Additionally, the intervention group of nurses measured significantly higher mean MAAS scores at the second week versus the comparison group (beta coefficient = 0.605, *p*-value < 0.001). See Table 4.

**Table 4:** Multivariable generalized linear mixed model regression explaining the nurses mean MI throughout the study time (N = 400).

Predictor		95% CI for (β) coefficient			
		*Beta (β) coefficient*	Lower	Upper	*p*-value
Intercept		1.34	0.67	2.01	** < 0.001**
		0.011	0.0001	0.022	**0.041**
Gender	Female, Male ^(ref)^	−0.04	-0.156	−0.006	**0.033**
Group	Intervention, comparison ^(ref)^	−0.027	−0.166	0.112	0.706
Time	4th week	1.138	0.903	1.373	< **0.001**
	3rd week	0.213	0.072	0.354	**0.003**
	2nd week	−0.157	−0.335	0.02	0.082
Interaction	Time*Group = Week3 * Intervention group	0.343	0.099	0.586	**0.006**
	Time*Group = Week2 * Intervention group	0.738	0.392	1.085	< **0.001**
	Time*Group = Week1 * Intervention group	0.605	0.34	0.869	** < 0.001**

Dependent variable: The nurses mean mindfulness intervention (MAAS) score; Link function: identity; *P* value significant: bold; Ref: reference group.

### The effect of MI on SD

Perceived sleep components were scored at baseline, before the nurses were assigned to the intervention or comparison groups. The participants' sleep quality was assessed and received an average score of 1.25 out of 3 points. The sleep latency, or the time required to fall asleep, was measured and yielded a mean score of 2.24 out of 3 points. Problematic sleep duration, referring to the length and quality of sleep, received a mean score of 0.68 out of three points. Participants also reported difficulty having efficient sleep with an average score of 0.71 out of 3 points. SD was common as indicated by a mean score of 1.68 out of 3 points. The use of sleep aid medications was relatively low, with an average score of 0.56 out of 3 points. Finally, daytime dysfunction due to poor sleep was assessed and received a mean score of 1.37 out of 3 points.

The nurses' mean perceived PSQI total score at baseline was 8.50 out of 21 total points with a standard deviation of 3.97 points. A higher PSQI total score implied greater perceived sleep problems. The nurses' top SD aspects at baseline were sleep latency, sleep disturbances, and daytime dysfunction due to poor subjective sleep quality. The second column of the table displays the nurses' mean PSQI score and its subscale scores at follow-up one month later for all nurses (N = 100). See Table 5.

**Table 5:** Descriptive analysis of the nurses perceptions of the PSQI scale seven main components at baseline and follow up times (One month apart). (N = 100).

	At baseline	One month later
Component1: Subjective sleep quality	1.25 (1.04)	0.71 (0.69)
Component-2: Sleep Latency	2.24 (1.49)	0.47 (0.72)
Component 3: Sleep duration	0.68 (0.78)	0.12 (0.33)
Component 4: Sleep efficiency	0.71 (0.97)	0.56 (0.78)
Component5: Sleep disturbance	1.68 (0.50)	1.14 (0.35)
Component 6: Use of sleep medications	0.56 (0.69)	0.38 (0.57)
Component-7: Daytime dysfunction	1.37 (1.25)	0.85 (0.94)
Global PSQI Score at baseline time	8.50 (3.97)	4.23 (2.39)

The greater scores on those seven components imply worse sleep quality.

Nurses' age showed a significant and negative correlation with mean PSQI score. Older nurses had significantly lower mean problematic sleep PSQI scores (beta coefficient = −0.029, *p*-value = 0.011). Ever-married nurses had a significantly higher mean problematic PSQI sleep quality score versus never-married nurses (beta coefficient = 0.165, *p*-value = 0.047). However, the overall mean perceived PSQI score at follow-up time (one month later) was significantly lower than at baseline, thus indicating a significant decline in nurses' problematic sleep PSQI score over time (beta coefficient = −0.594, *p*-value < 0.001). The interaction effect of the MI with time (intervention*time) was not statistically significant, thus suggesting that the decline in sleep problems in the intervention group may not differ significantly from the comparison group (*p*-value = 0.067). The multivariable adjusted findings suggest that nurses working night shifts may have slightly lower mean perceived problematic sleep scores relative to nurses working other shift types, although this difference was not statistically significant (p-value = 0.070). See Table 6.

**Table 6:** Multivariable generalized linear mixed model regression explaining the nurses mean perceived (PSQI) score throughout the study time. (N = 200).

Predictor		95% CI for (β) coefficient			
		*Beta (β) coefficient*	Lower	Upper	*P*-value
Intercept		3.554	2.683	4.424	** < 0.001**
Age-years		−0.029	−0.051	−0.007	**0.011**
Gender	Female	−0.069	−0.244	0.106	**0.436**
Marital state	Ever married	0.165	0.002	0.328	**0.047**
Mean perceived sufficiency of HH Income score		0.139	0.05	0.0227	**0.002**
Nurse Group	Intervention	−0.237	−0.494	0.021	**0.071**
Time	One month later	−0.594	−0.767	−0.421	** < 0.001**
Interaction effect	(Time*Group) = One month later*Intervention group	−0.242	−0.501	0.108	**0.067**
Type of worked shifts = C- Night shifts		−0.277	−0.577	0.022	**0.07**

Dependent variable = The nurses mean perceived (PSQI) sleep quality score. Link function: identity. *P* value significant: bold.

## Discussion

The results found worse levels of SD among Jordanian nurses in intensive care units. Other studies showed similar results where nurses had low levels of sleep quality and high level of SD^17–19^. Sleep quality is often tied with the ability of individuals to achieve a restful state within a conducive environment; hence, nurses working in clinical environments with high pressures and demands emanating from the nature of patient work can find it difficult to achieve good sleep quality^20,21^. For instance, nurses find it hard to achieve enough sleep when shifts are long (i.e., 12-h shifts), when shifts are fixed on non-social hours (i.e., continuous night shifts), and when shifts are highly variable with inadequate rest/off days in between^22^.

In addition, nurses are left with less time to spend on sleep and thus experience SD when they have early start times but leave late due to high pressures from work such as nurse shortage, high patient acuity, and high task load^23^. Moreover, increased SD was associated with nurses who have a lack of bathroom needs, abrupt wakeups, bad dreams, and difficulty breathing from stress or other non-fatal physiological reasons. No sociodemographic factors were significantly related with SD. However, a significant negative relationship was found between sleep quality and compassion fatigue. Nurses who had high levels of STS reported significantly high SD.

The better sleep quality among married nurses seen here might be hypothesized by the presence of social support received by nurses from their partners; however, this hypothesis will need to be tested in future research^24^. This study also showed that individuals with better socioeconomic status reported better sleep quality and duration than individuals with worse socioeconomic status. This result might be because individuals with worse socioeconomic status reported constant worry and anxiety arising from their worse financial and economic conditions, thus reducing their ability to gain good sleep quality and length of sleep^25–27^.

The results showed that the implementation of the MI significantly reduced levels of SD similar to the results of other studies that explored the values and benefits of mindfulness-based interventions for healthcare professionals^28–30^. The rationale for the abovementioned studies in exploring the benefit of mindfulness for healthcare professionals was that mindfulness was shown^28,31–33^ to positively benefit psychological and physical conditions as well as the coping mechanisms of people faced with stressful situations. In addition, the components of sleep quality (i.e., subjective sleep quality, sleep latency, sleep duration, sleep disturbance, use of sleep medications, and daytime dysfunction) improved among nurses who underwent mindfulness-based intervention.

The prolonged benefit of mindfulness points to its usability as a tool to improve physical and psychological conditions of nurses working in acute care settings^28,31–33^. Statistical analyses from such studies demonstrated the sustained benefits of mindfulness in improving psychological outcomes over time. A systematic review by Kriakous and colleagues^34^ explored the effectiveness of mindfulness-based stress reduction on the psychological functioning of healthcare professions and recommended utilizing mindfulness to support nurses and other healthcare professions in their work.

Videos about mindfulness were watched by the participants in the comparison group as recommended by a certified expert with extensive experience in conducting mindfulness interventions. This video intervention is also more ethically acceptable for comparison groups versus doing nothing. It is also more convenient and feasible to watch these videos after their busy working hours. We also considered that the effect of watching mindfulness videos mimics the effect of nonspecific active controls used for time/attention-matched interventions to control for placebo effects as shown in a recent systematic review^35^. This systematic review found that sleep quality was improved after mindfulness meditation interventions versus nonspecific active controls including at 5- to 12-month follow-up in both groups. This improvement in both groups is consistent with our results pertaining to both groups (MI sessions vs. watching videos about mindfulness). Here, the improvements in sleep quality in the intervention group had a bigger effect size than the comparison group at the end of month.

Recent numerous reports have also demonstrated the same effect of MI with various protocol durations. These results showed that MI significantly enhances sleep quality and reduces sleep disturbance based on the variant frequencies of the MI^36–39^, e.g., twice a week for more than three months with total time duration more than 24 h^36^; an eight-week MI program^38^; or a 6-week MI for two hours per week^39^. Therefore, our findings are consistent with findings of the prior work^36–39^ and revealed significant improvements in sleep quality after conducting MI versus control groups. This study recruited a smaller sample size than Zhou and colleagues' study^37^, which used an online survey. However, that study was a randomized clinical trial with face-to-face interviews that measured sleep quality for two parallel groups of homogenous characteristics of participants including age, gender, education, and work experience.

In this study, the research design was controlled by performing MI by the certified expert for all participants in the intervention group in contrast to work using mindfulness-based interventions to reduce stress and promote sleep quality^38^. In that work, participants were given written material and CDs with meditation guides to perform the intervention at home on a daily basis. Moreover, the current study reduces the threat to the external validity through conducting the MI in two settings, in which the principal investigator evaluated the sleep quality in-person with participants. This methodology is in contrast to what was conducted in a randomized control trial^39^ that used a single-site and participants completed self-reports of sleep quality.

In the current study, all participants adhered to complete all MI sessions due to sending frequent regular reminders via text messages by the PI. However, the attrition rate as a challengeable issue in MI studies was discussed in previous recent studies^40,41^. The attrition rate of mindfulness-based cognitive therapy was high among participants with health issues, such as cognitive reactivity, brooding, and depressive rumination^40^. In a recent meta-analysis^41^, the weighted mean attrition rate was 19.1% across 114 studies, revealing that MIs might be less acceptable among participants than alternative interventions.

## Limitations

There are some limitations in our study. First, the larger sample size might be considered more robust in providing more accurate results in measuring the changes in mindfulness between intervention and control groups. Future studies targeting the MI might be more focused on longitudinal research design to capture the time-serial changes of mindfulness among participants. Eventually, longer protocol duration of MI sessions might significantly have a stronger effect on the participants to enhance the effectiveness of MIs on measured outcomes of the future studies.

## Conclusion

Nurses reported significantly high levels of SD. The MI was found to significantly reduce the level of the SD and improve the sleep quality among nurses. Strategies to implement MI should be incorporated into nursing orientation and development programs at hospitals to counter the adverse effects of the SD. Nursing administrators should provide support to strategies, interventions, and initiatives to promote MI among nurses.

## Data Availability

Date can be requested from the first author (Audai A. Hayajneh) upon a reasonable request.
